# Cost of Tuberculosis Care in Programmatic Settings from Karnataka, India: Is It Catastrophic for the Patients?

**DOI:** 10.1155/2020/3845694

**Published:** 2020-05-11

**Authors:** M. P. Poornima, M. N. Shruthi, Ashwini Laxmanrao Chingale, V. Veena, Sharath Burugina Nagaraja, Akshaya Kibballi Madhukeshwar

**Affiliations:** ^1^Department of Community Medicine, Jagadguru Jayadeva Murugarajendra Medical College, Davangere, Karnataka, India; ^2^Department of Community Medicine, BGS Global Institute of Medical Sciences, Bengaluru, Karnataka, India; ^3^Department of Community Medicine, Belagavi Institute of Medical Sciences, Belagavi, Karnataka, India; ^4^Department of Community Medicine, Employees State Insurance Corporation Medical College and Post Graduate Institute of Medical Sciences and Research, Bengaluru, Karnataka, India; ^5^Department of Community Medicine Yenepoya Medical College, Yenepoya University, Mangaluru, Karnataka, India

## Abstract

**Background:**

TB diagnostic and treatment services in India are provided free of cost in the programmatic context across the country. There are different costs incurred during health care utilization, and this study was conducted to estimate such costs. *Methodology*. A longitudinal study was conducted among patients of three urban tuberculosis units (TUs) of Davangere, Belagavi, and Bengaluru, Karnataka. Trained data collectors administered a validated questionnaire and recorded monthly costs incurred by the patients which are expressed in median Indian National Rupees (INR). The analysis was done using SPSS version 23.0. A *p* value of <0.05 was taken as statistically significant.

**Results:**

Among 214 patients, about 37%, 42%, and 21% belonged to Davangere, Belagavi, and Bengaluru, respectively. Median total pre- and postdiagnostic costs incurred across the three TUs were 3800 and 4000 INR, respectively. The direct nonmedical cost was higher for accommodation (median cost of 800 INR) and direct medical cost for non-TB drugs (median cost of 2000 INR). However, maximum direct medical and nonmedical costs were attributed to hospital admissions (1200 INR) and accommodation costs (700 INR) in the postdiagnostic period, respectively. The median indirect cost incurred was 300 INR overall, and the maximum total indirect cost was 40000 INR in the postdiagnostic period. About one-third of patients faced loss of income and 19.6% faced coping costs. Patients spent about 6.7% (0.97%–52.3%) of their income on TB treatment. About 12.3% patients faced catastrophic expenditure. Median cost was significantly higher among those seeking private health care facilities (12100 INR in private vs. 6800 INR in public; *p* < 0.05) during the prediagnostic period. Prediagnostic and diagnostic out-of-pocket expenditures (OPE) were significantly higher across all the three centres (*p* < 0.05).

**Conclusion:**

The TB patients experienced untoward expenditure under programmatic settings. The costs encountered by one in eight patients were catastrophic by nature.

## 1. Introduction

Tuberculosis (TB) ranks among the top ten causes of death worldwide and is the leading cause from a single infectious agent. Globally, 10.0 million (range, 9.0–11.1 million) new cases of TB were reported in 2017. There were 2.8 million cases and 410,000 deaths in India during 2018 which accounted for about a quarter of the world's TB in terms of both incidence and mortality. TB affects individuals in their most economically productive age, adding to the social and economic disruption [1, 2]. Karnataka is one of the high HIV-TB burden states. The total default rate and death rate have decreased to 7% and 6% in 2015 despite the high HIV-TB burden, respectively.

The Revised National Tuberculosis Control Programme (RNTCP) is the world's largest TB control programme launched in 1997 to achieve TB control in India. It provides countrywide accessibility of free TB diagnostic and treatment services to the population. Despite this provision from the public health sector, 60 to 88% of Indian patients seek TB care from the private health sector, and around 90% of them prefer to buy anti-TB drugs from the private market irrespective of their financial status [3]. The TB diagnostic practices in the private sector differ from those in the public sector wherein the primary emphasis under RNTCP is on sputum and chest radiography examination. Utilizing the private sector for additional diagnostic services might add to the costs incurred by the TB patients [4].

There are various hardships that can arise during health care utilization due to different costs incurred, viz., direct medical and nonmedical and indirect costs such as loss of income despite free TB diagnostic and treatment services [5]. Such out-of-pocket costs for public healthcare services may lead to the “‘medical poverty trap,” wherein the TB disease per se might lead to poverty and also might exacerbate the existing poverty of the already poor [6]. Though aggregate real costs might appear lesser for poor people, relative costs as a percentage of their income are much higher [7, 8].

TB programmes therefore have to ensure that patients do not face economic barriers that keep them from seeking timely diagnosis and treatment and adherence to the treatment [9]. Thus, assessing the cost incurred by the affected TB families can act as a baseline to take further actions and interventions and serve towards achieving zero catastrophic cost which is one of the goals of the End TB Strategy [10]. In addition to it, there is paucity in the literature on follow-up studies conducted on the costs incurred by the TB patients in the urban settings. We conducted a study to estimate the total, direct, and indirect costs incurred by TB patients in urban areas for diagnosis and treatment of TB registered with RNTCP across three urban tuberculosis units in the state of Karnataka, India.

## 2. Materials and Methods

**Figure 1:**
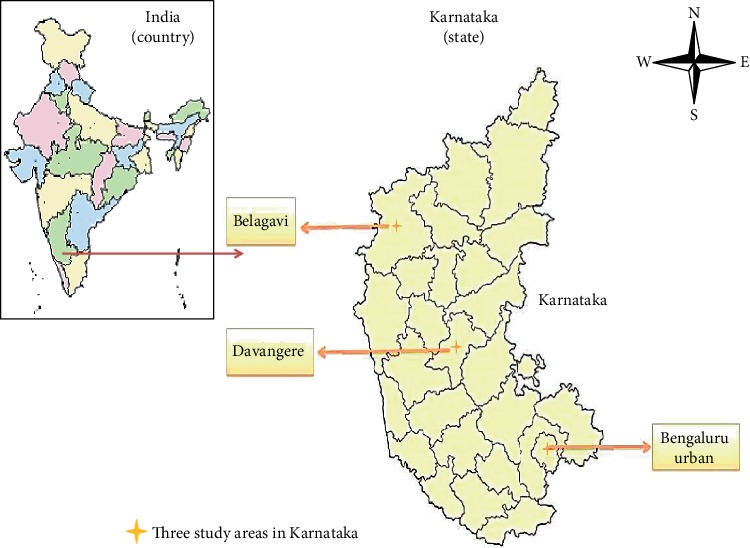
Map of the three urban TUs which were selected for the study in the state of Karnataka, India (2017-2018**)**.

A longitudinal study was conducted in Karnataka during May 2017 through March 2018. Karnataka, a southern state in India with a population of 67.7 million, is the eighth largest state comprising 30 administrative districts [11]. The total literacy of the state is nearly 75%, and about 34% of the population resides in urban areas. For the purpose of this study, we selected three districts from the northern, central, and southern regions, namely, the Davangere, Belagavi, and Bengaluru urban districts (Figure 1). We selected one urban TU from each of these districts. A TB unit (TU) is a subdistrict supervisory unit, and it usually serves a population of 0.15 million to 0.25 million in urban areas. Davangere district has one urban TU catering to a 0.45 million population, Belagavi district has one urban TU catering to a 0.52 million population, and Bengaluru urban district has 14 urban TUs catering to a 5.4 million population. In the Bengaluru urban district, we selected the Kengeri TU purposively which caters to a 0.18 million population. All the three TUs have a medical college in their vicinity, and faculty from each of these colleges served as the investigator in this study [12].

### 2.1. Study Population

All the new and previously treated patients registered under RNTCP from May 2017 to July 2017 were part of this study. We included patients who had completed at least one month of antitubercular treatment. It was mainly to ensure and ascertain that the selected patients were residing in the same area for further follow-up. The new and previously treated TB cases were followed up for a period of 6 months and 8 months, respectively. Patients taking treatment from the private sector but registered on Nikshay (a web-based TB recording and reporting system in India) were excluded from the study. Patients lost to follow-up, who transferred out, and who died during the course of treatment and proven drug-resistant TB cases were excluded from analysis. Written informed consent was obtained from the patients before initiating the study, and ethics clearance was obtained from the Institutional Ethics Committees of Jagadguru Jayadeva Murugarajendra Medical College, Davangere; Belagavi Institute of Medical Sciences, Belagavi; and BGS Global Institute of Medical Sciences, Bengaluru. The requisite permission was obtained from the state TB office, Karnataka, to conduct the study.

### 2.2. Data Collection, Variables, and Sources of Data

**Figure 2:**
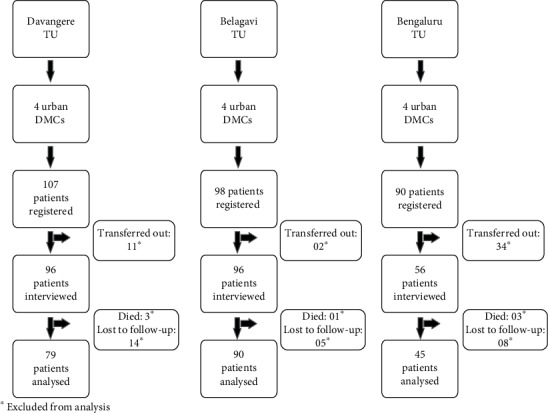
Flow chart representing the selection of patients for the study in the state of Karnataka, India (2017-2018) (*N* = 214).

A line list of all the eligible TB patients from the three TUs was prepared (Figure 2). The medicosocial workers (MSWs) from each medical college were trained for data collection using a pretested semistructured questionnaire after customization to the local settings [9]. The questionnaire had two parts: part one was to elicit the information on the sociodemographic profile and prediagnostic and diagnostic costs and part two on treatment costs and expenditure during follow-up visits. The part one of the questionnaire was administered by the MSWs to all the patients at the first point of contact, while the part two section of the questionnaire was given to the patients to carry home and was explained in detail in their local vernacular language (Kannada) that they were to enter the monthly expenditure incurred for any events related to TB care. The MSWs interacted with patients at regular intervals either by visiting patients' homes or talking to them over the phone and monitored the entries made in the questionnaires every month. The investigators validated the data randomly once in three months. The operational definitions have been considered for few variables in this study and are listed in Table 1.

### 2.3. Data Entry and Statistical Analysis

All the collected data were entered into Microsoft excel. The continuous variables were expressed as mean and standard deviation while the categorical variables were expressed as proportions. The costs incurred are expressed as median and range. The cost incurred was expressed in Indian National Rupees (INR) (one American US dollar was equivalent to 63.67 Indian rupees as on January 2018) [17]. The differences between the total costs incurred among different sociodemographic variables were analysed using a Mann-Whitney *U* test for comparing two categories and a Kruskal-Wallis test for comparing more than two categories. A chi-square test was used to determine the association of health care-seeking behavior with the total cost incurred. The median OPE cost incurred in the prediagnostic and diagnostic periods was compared with the postdiagnostic period using a Wilcoxon signed rank test. The modified BG Prasad classification was used to assess the socioeconomic status [18]. Statistical analysis was done using the IBM Statistical Package for Social Sciences (SPSS) for Windows, version 23.0. A *p* value of <0.05 was considered as statistically significant.

## 3. Results

A total of 214 patients were enrolled for the study, of which, 37% (79), 42% (90), and 21% (45) were from the TUs of the Davangere, Belagavi, and Bengaluru districts, respectively. The median age of the study participants was 36 years (24–49 years). The majority (62%) of the patients were males, 36% were unemployed, and 71% belonged to below the poverty line. Twelve (5.6%) of them were HIV positive. Most of them belonged to the class III socioeconomic status according to the modified BG Prasad classification updated for base March 2018. The majority (94%) of the cases were newly diagnosed and had pulmonary TB (69%). Nearly 58% of patients had sought a public health care facility predominantly (Table 2).

### 3.1. Direct and Indirect Costs Incurred during Prediagnostic Period

The details of cost incurred during the prediagnostic period across the three TUs are shown in Table 3. The total prediagnostic cost incurred by the TB patients in the study was 3800 INR (highest of 5600 INR from Bengaluru). The direct costs for medical and nonmedical were found to be 5000 and 3000 INR, respectively. The indirect cost incurred was 300 INR.

### 3.2. Direct and Indirect Costs Incurred during Postdiagnostic Period

The details of cost incurred during the postdiagnostic period across the three TUs are shown in Table 4. The total postdiagnostic cost incurred by the TB patients in the study was 4000 INR (highest of 5000 INR from Bengaluru). The direct costs for medical and nonmedical were found to be 800 and 700 INR, respectively. The indirect cost incurred was 400 INR.

The mean total time spent in transportation and at waiting at the clinic or hospital during prediagnosis and diagnosis was 105.66 ± 42.86 minutes (excluding those admitted in the hospital for diagnosis where they spent days ranging between 1 and 5 days for diagnosis). During follow-up, mean total time spent in transport and at the clinic or hospital postdiagnosis was 85.5 ± 41.07 minutes (excluding those admitted in the hospital for reasons, viz., generalized weakness, effusion tapping, hepatitis, and fever where they spent days ranging between 1 and 3 days in hospitalization). The majority of our study participants (184, 86%) spent for health supplements (protein powders/vitamins/eggs/fruits/nonvegetarian food). About 32% (69) faced loss of income due to either loss of wages or loss of job, and 20% (42) faced coping costs. Patients spent about 6.7% (range 0.97%–52.3%) of their income on TB treatment. About 12% of patients had spent more than ten percent of their average monthly income.

Among the sociodemographic variables, patients of the younger age group (≤36 years), males, extrapulmonary TB, those seeking a private health care facility, and the class III socioeconomic group faced a higher median cost compared to the other groups. Costs were significantly higher and nearly double among those seeking a private health care facility (12100 INR in the private sector against 6800 INR in the public sector; *p* < 0.05). Among these factors, health-seeking behavior was significantly associated with a higher median OPE cost of 8000 INR (*p* < 0.05). (Table 5).

The prediagnostic and diagnostic OPE cost incurred by the TB patients was significantly higher compared to the postdiagnostic OPE cost across all the three centres (*p* < 0.05) (Table 6).

## 4. Discussion

This is one of the first studies conducted in Karnataka to estimate the financial burden on TB patients under programmatic settings. The study is pertinent as the state has a dual burden of TB/HIV and poverty [19]. Our study findings revealed that TB patients incur considerable costs and financial losses before and during their TB treatment although the services are made freely available at public health care facilities. Comparatively, the extrapulmonary TB patients had spent more during their prediagnostic period in the private sector. The majority of patients belonged to the productive age group (15-30) with more than 60% being males and also employed. TB is presumed to be a disease of the poor in India and was evident in the current study where nearly 70% were falling below the poverty line and belonged to the middle, lower middle, and lower classes (73.4%) [20]. The public health care facility was predominantly (58%) sought as a first point of care in our study.

The total prediagnostic cost incurred by the TB patients in our study was 3800 INR, and the highest median cost incurred was reported in Bengaluru (5600 INR). The higher direct medical cost was incurred for non-TB drugs reflecting that patients have been treated empirically for the presented symptoms rather than being referred for TB diagnosis. Followed by non-TB drugs, the higher median cost was for laboratory investigations, and in such instances, the patients were subjected to diagnostic tests for extrapulmonary TB in a private health care facility like FNAC (fine needle aspiration cytology), biopsy, ultrasonography, and HRCT (high-resolution computed tomography). The higher range of diagnostic costs incurred by the patients treated in the Bengaluru setup could be due to the patients seeking varied channels of health care facilities like tertiary setups including corporate hospitals and also health-seeking behavior like opting for private setups with a belief of better quality care as compared to public health facilities [21]. It has also been observed that a sizeable number of patients during the prediagnostic period used the private services for diagnosis and later got transferred to a TB control programme [22]. Hence, ensuring efficient diagnostic algorithms and ensuring similar practices among private practitioners can reduce the financial burden of TB patients [23].

The costs incurred in the postdiagnostic period were mainly contributed by the purchase of health supplements and hospitalization. The health supplements are advised by the health care providers to improve the nutritional status of the TB patients though there are insufficient literatures existing [24]. Nutritional supplements are thought to help people recover from the illness by strengthening their immune system and by improving weight gain and muscle strength, allowing them to return to an active life [23]. Nearly 86% of the TB patients spent on health supplements (protein powders/vitamins/eggs/fruits/nonvegetarian food). Apart from that, the direct medical cost was high for hospitalizations which were due to the initial case management of TB patients. In this regard, a systematic review has found that hospitalization contributed to 74% of all drug-sensitive TB provider costs, and it was about 51% in lower middle income countries [25]. Observing the range of direct medical and nonmedical costs, overall, direct nonmedical cost seemed to be relatively high which is referred to as “hidden costs.” Direct medical cost seems to be relatively reduced as ultimately all the patients linked to the RNTCP are provided free treatment [26].

Patients also faced loss of wages and incurred coping costs (ranging from 0 to 15000 INR) due to TB which can be linked to the higher costs incurred and also by those belonging to the lower socioeconomic status. These findings are in line with the study by John et al., where TB patients faced substantial catastrophic costs associated with their illness, and the overall costs exceeded 193% of the estimated monthly income of daily wage labour [27]. There is substantial evidence showing financial burden of TB in middle- and low-income countries, where the average total costs incurred for TB treatment were equivalent to 58% of annual individual income and 39% of household income [28]. In this study, about 32.2% (69 patients) faced loss of income due to either loss of wages or loss of job and 19.6% (42 patients) faced coping costs. Coping strategies of households in our study included the sale of assets, taking up debt, saving on food or other items, and the patient attendant leaving a job or taking leave to care of the patient. Along with this, patients spent about 7% (range 1% to >50%) of household income on TB treatment. Around one in eight patients spent more than 10% of the average monthly income indicating financial impact and which was considered catastrophic in our study.

We found that the mean total time spent during transport and in the clinic or hospital during prediagnosis and diagnosis was 2 hours and 1.5 hours during follow-up which was considerably less when compared to other studies. The differences noted may be due to the consideration of different study settings, patient preferences in selecting the hospitals for utilization of health care, and location of DOTS centres [27].

Varied costs for different diagnostic services across the private facilities can explain the relatively higher cost at the private facilities. The care-seeking pattern, empirical treatment for non-TB, and investigations of higher cost for extrapulmonary TB like fine needle aspiration cytology, biopsy, ultrasonography, and computed tomography scans point towards incurring higher median OPE in the prediagnostic period. Though one-third of the patients sought health care from a private facility initially, all were later linked to public health facilities for the purpose of treatment which is implicated by the zero TB-related drug cost in the postdiagnostic period. This also explains the lesser postdiagnostic costs incurred in our study.

Under the Nikshay Poshan Yojana, all the patients with TB in India are provided with a cash incentive of 500 INR every month through the Direct Benefit Transfer to their bank accounts to address the financial and nutritional hardship the patient and family undergoes due to TB and to reduce catastrophic costs to the patient [10, 28]. The linkage of the TB patients under each DOTS centre to the nearby grocery stores for provision of dry rations using the untied funds of hospital management committees may also be thought of as an option over and above the amount transferred to TB patients under the DBT scheme [29].

Recently launched, Pradhan Mantri Jan Arogya Yojana is aimed at providing health insurance cover of up to 5 lakhs INR per family per year and is intended to significantly reduce out-of-pocket expenditure for hospitalization and mitigate financial risk arising out of catastrophic health episodes [30].

Our study has the following strengths and limitations. The strengths are as follows: (1) As it is a multicentric study conducted across three different urban settings of Karnataka, it reflects the scenario of urban areas of the state. (2) The longitudinal study design with regular follow-up improved the quality of data and reduced the recall bias especially when it comes to the amounts spent by the patients. The limitations are as follows: (1) We have determined the cost based on a prevalent approach that measures costs for an episode of illness, and hence, the lifetime cost of TB illness could not be determined. (2) The study participants had only TB patients taking treatment from a public health facility, and the estimated costs might be lower than the actual costs. (3) Validation of the coping costs expended towards TB by selling of assets, borrowing, etc. was not possible.

## 5. Conclusion

One in eight TB patients encountered catastrophic costs for TB care under programmatic settings. Comparatively, the untoward expenditures were seen more before the diagnosis. Decentralizing efficient diagnosis and treatment within the reach of patients can minimize the patient costs. Focus on a patient-centric approach with effective reimbursement mechanisms and utilizations of social insurance initiatives is vital to reduce patients' out-of-pocket expenditures.

## Figures and Tables

**Table 1:** Operational definitions used in the study.

(1) Total prediagnostic and diagnostic costs: it includes any costs incurred directly or indirectly before the diagnosis and during the diagnosis of TB (namely, any laboratory investigations and costs towards diagnosis of other morbidity)
(2) Total postdiagnostic and treatment costs: it includes any costs incurred directly or indirectly for the purpose of treatment or follow-up or loss of wages by the patients
(3) Direct medical costs: it includes any cost incurred on consulting a doctor and the expenditure made on investigations and drugs including expenses on non-TB drugs
(4) Direct nonmedical cost: it includes costs incurred towards travel, accommodation, and special food for the patient and the accompanying personnel
(5) Indirect costs: it includes costs due to loss of wages of the patient due to illness and debilitating condition leading to decreased capacity to work thus necessitating change in the type of work performed
(6) Total cost: it comprises expenditure during the pretreatment and the treatment phase under direct and indirect cost categories
(7) Coping cost: these are the costs that are incurred through coping strategies of a household to meet daily requirements despite the extra expenditures or loss of income. It includes the sale of fixed or moveable assets, loans, saving on food or other items, the patient attendant leaving a job/taking leave to care for the patient, taking a child out of school to care for the patient, or taking up another job [9, 13, 14]
(8) Out-of-pocket expenditure (OPE): it includes direct health expenditures on diagnosis and treatment (consultation fees, laboratory investigations, radiography, drugs, and hospital charges for services) and associated nonmedical expenses (transport and accommodation costs for patient and attendants and nutrition supplement costs) but not income loss [15]
(9) Catastrophic cost: if more than 10% of the average monthly income is spent/claimed to be spent as TB-related expenditures, then it is considered catastrophic [16]

**Table 2:** Sociodemographic characteristics of the TB patients (*n* = 214) in the three selected urban TB units of Karnataka, India (2017-2018).

Variables	Davangere	Belagavi	Bengaluru	Total
	(n1 = 79)	(n2 = 90)	(n3 = 45)	(n = 214)
*Age (median (IQR)) in years*	40 (23-51)	37.5 (27-47)	31 (27-47)	36 (24-49)
*Age group in years*				
<14 yrs	00 (0.0)	03 (3.3)	03 (6.7)	06 (2.8)
15-30 yrs	28 (35.9)	29 (32.2)	19 (42.2)	76 (35.5)
31-45 yrs	29 (37.2)	33 (36.7)	10 (22.2)	72 (33.6)
46-60 yrs	10 (12.8)	17 (18.9)	11 (24.4)	38 (17.7)
>60 yrs	12 (15.4)	08 (08.9)	02 (4.4)	22 (10.2)
*Gender (%)*				
Males	53 (67.1)	52 (57.8)	28 (62.2)	133 (62.1)
Females	26 (32.9)	38 (42.2)	17 (37.8)	81 (37.8)
*Occupation (%)^¥^*				
Employed	47 (59.4)	53 (58.8)	31 (79.5)	131 (61.2)
Unemployed	32 (40.5)	37 (41.1)	08 (20.5)	77 (35.9)
*Patients below poverty line (%)*	58 (73.4)	69 (76.7)	25 (55.6)	152 (71.0)
*Socioeconomic status^ᴥ^ (%)*				
Class I	08 (10.1)	07 (7.7)	12 (26.7)	27 (12.6)
Class II	13 (16.5)	13 (14.4)	21 (46.7)	28 (13.0)
Class III	20 (25.3)	31 (34.4)	12 (26.7)	63 (29.4)
Class IV	21 (26.6)	28 (31.1)	0	49 (22.8)
Class V	17 (21.5)	11 (12.2)	0	28 (13.0)
*HIV-positive status*	02 (2.53)	10 (11.1)	0	12 (5.6)
*Type of TB (%)*
Pulmonary	59 (74.6)	61 (67.8)	28 (62.2)	148 (69.1)
Extrapulmonary	20 (25.3)	29 (32.2)	17 (37.8)	66 (30.8)
*TB category (%)*				
New cases	74 (93.7)	84 (93.3)	44 (97.8)	202 (94.3)
Previously treated	05 (06.3)	06 (06.7)	01 (02.2)	12 (5.6)
*Health-seeking behavior (%)*				
Public health care facility	53 (67.0)	51 (56.7)	20 (44.5)	124 (57.9)
Private health care facility	13 (16.4)	34 (37.8)	25 (55.5)	72 (33.6)
Traditional healer	07 (08.8)	0	0	07 (3.2)
Pharmacy	06 (07.5)	05 (5.6)	0	11 (5.1)

^¥^Children and students were excluded (*n* = 6) in Bengaluru. ^ᴥ^Modified BG Prasad classification was used.

**Table 3:** Direct and indirect costs incurred during prediagnostic periods in the three selected urban TB units of Karnataka, India (2017-2018).

Variables	Median (range) in INR			
	Davangere (n1 = 79)	Belagavi (n2 = 90)	Bengaluru (n3 = 45)	Total (n = 214)
Prediagnostic & diagnostic cost	2200 (400-15000)	2000 (500-10000)	5600 (500-82500)	3800 (400-82500)
(a) Direct cost: medical	1500 (0-7000)	900 (0-5000)	1500 (0-70000)	5000 (0-70000)
Administrative cost	200 (0-500)	200 (0-650)	300 (0-20000)	200 (0-20000)
Laboratory investigations^¥^	400 (0-5000)	450 (0-4000)	800 (0-50000)	500 (0-50000)
Non-TB drug cost^§^	450 (0-850)	500 (0-900)	0 (0-10000)	2000 (0-10000)
(b) Direct cost: nonmedical	1900 (0-8000)	1300 (0-4500)	1200 (0-15000)	3000 (0-15000)
Transport	350 (0-550)	300 (0-650)	480 (0-5000)	440 (0-5000)
Food	180 (0-600)	200 (0-400)	300 (0-2500)	250 (0-2500)
Accommodation^§^	0 (0-1000)	0 (0-900)	0 (0-5000)	800 (0-5000)
Guardian cost	200 (0-450)	200 (0-450)	600 (0-4000)	400 (0-4000)
(c) Indirect	200 (0-500)	150 (0-600)	200 (0-800)	300 (0-800)

^§^As majority had zero cost, median cost is zero. ^¥^Sputum, X-ray, CT scan, magnetic resonance imaging, fine needle aspiration cytology, ultrasound-guided biopsy, and bronchoalveolar lavage.

**Table 4:** Direct and indirect costs during postdiagnostic periods and coping costs incurred by TB patients in the three selected urban TB units of Karnataka, India (2017-2018).

Variables	Median (range) in INR			
	Davangere (n1 = 79)	Belagavi (n2 = 90)	Bengaluru (n3 = 45)	Total (n = 214)
Postdiagnostic costs	800 (400-18500)	700 (300-21500)	5000 (850-41900)	4000 (300-41900)
(a) Direct cost: medical	800 (0-5800)	600 (0-3000)	1040 (0-6000)	800 (0-6000)
Consultation charges^§^	0 (0-450)	0 (0-400)	0 (0-2000)	100 (0-2000)
Laboratory investigations^¥§^	0 (0-1300)	0 (0-900)	0 (0-2900)	500 (0-2900)
Drug cost	0 (0-900)	0 (0-1500)	0	0
Health supplements^ᴥ^	400 (0-2000)	450 (0-1800)	600 (0-6000)	400 (0-6000)
Administrative cost due to hospitalization^§^	0 (0-1500)	0 (0-2000)	0 (0-14000)	1200 (0-14000)
(b) Direct cost: nonmedical	800 (0-1500)	1200 (0-2500)	1500 (0-9700)	700 (0-9700)
Transportation	350 (0-700)	250 (0-500)	650 (0-2700)	500 (0-2700)
Food	200 (0-400)	300 (0-700)	650 (0-2300)	450 (0-2300)
Accommodation^§^	00 (0-450)	00 (0-700)	0 (0-3000)	700 (0-3000)
Guardian cost	150 (0-250)	200 (0-500)	180 (0-3000)	200 (0-3000)
(c) Indirect	500 (0-8000)	300 (0-10000)	0 (0-40000)	400 (0-40000)
Total indirect: foregone income^§^	0 (0-8000)	0 (0-10000)	0 (0-40000)	2000 (0-40000)
Coping costs^§^	0 (0-15000)	0 (0-10000)	0 (0-10000)	1000 (0-15000)

^§^As majority had zero cost, median cost is zero. ^¥^Sputum, X-ray, CT scan, magnetic resonance imaging, fine needle aspiration cytology, ultrasound-guided biopsy, and bronchoalveolar lavage. ^ᴥ^Protein powders/vitamins/eggs/fruits/nonvegetarian food.

**Table 5:** Comparison of total cost incurred by the TB patients among various sociodemographic groups in the three selected urban TB units of Karnataka, India (2017-2018).

Variables	Median (IQR)	*N*	Mean rank	*U* value/*H* value	*p* value
*Median age in years^ᴥ^*					
≤36	10320 (9287.5)	24	24.94	*U* = 205.50	0.29
>36	8550 (9410.0)	21	20.79		
*Gender^ᴥ^*					
Males	10680 (7733.8)	28	24.95	*U* = 183.50	0.20
Females	7800 (6495.0)	17	19.79		
*Type of TB^ᴥ^*					
Pulmonary	8075 (8810.0)	28	20.33	*U* = 162.50	0.078
Extrapulmonary	10000 (17925)	17	27.44		
*Health seeking behavior* ^∗^					
Public health care facility	6800 (5882.5)	20	16.28	*U* = 115.5	0.002^∗^
Private health care facility	12100 (10975)	25	28.38		
*Socioeconomic status^¥^*					
Class I	900 (17742.5)	12	23.29	*H* = 2.25	0.33
Class II	7800 (7935.0)	21	20.31		
Class III	11325 (6218.75)	12	27.42		

^∗^Indicates significant difference at *p* < 0.05. ^ᴥ^Mann-Whitney *U* test. ^¥^Kruskal-Wallis test. Note: health-seeking behavior was also significantly associated with the higher median out-of-pocket expenditure (OPE), i.e., ≥8000 INR on applying a chi-square test (*χ*^2^ value being 24.80 and *p* < 0.001).

**Table 6:** Comparison of median prediagnostic and diagnostic with postdiagnostic out-of-pocket expenditure (OPE) incurred among the TB patients in the three selected urban TB units of Karnataka, India (2017-2018).

Study TB units	Prediagnostic and diagnostic OPE Median (range) INR	Postdiagnostic OPE Median (range) INR	*Z* value^ᴥ^ (*p* value)
Davangere	2200 (400-15000)	800 (400-18500)	-3.42 (0.00)^∗^
Belagavi	2000 (500-10000)	700 (300-21500)	-3.86 (0.00)^∗^
Bengaluru	5600 (500-82500)	5000 (850-10520)	-2.02 (0.04)^∗^
Total	3800 (400-82500)	4000 (300-21500)	-3.10 (0.02)^∗^

^∗^Indicates a significant statistical association with *p* < 0.05. ^ᴥ^Wilcoxon signed rank test has been applied.

## Data Availability

data are not freely available, there is concern for patients privacy and as it is collected among patients registered to govt facility , sharing of data may have legal issues.
